# A Model Hospital at the Cartwright Exhibition, Bradford

**Published:** 1904-06-04

**Authors:** 


### A MODEL HOSPITAL AT THE CARTWRIGHT EXHIBITION, BRADFORD.

At this Exhibition, which was opened by the Prince and Princess of Wales on May 4th, an interesting exhibit was a model hospital built by Messrs. Humphreys, of Knightsbridge, and furnished by Messrs. Reynolds and Branson, of Leeds, with hospital furniture, appliances, instruments, etc. The operation-room contained an operating table fitted with hot-water chambers, head and foot levers, etc.; a new soiled-dressing bucket, made of enamelled iron with nickel-plated lid, the lid raised by means of a foot lever; the latest pattern of Schimmelbusch's steriliser, an electric steriliser for instruments and dressings, lotion bowls, instrument cabinet containing instruments, stand irrigator with adjustable rod, aseptic tables, Doulton's latest pattern lavatory basin, etc. In a room adjoining the hospital Messrs. Reynolds and Branson, Limited, have a complete installation of *x*-ray and electro-therapeutic apparatus, the *x*-ray coil being worked from the main current of 230 volts through one of their portable resistance tables which reduces the current also to suit cautery, motor drill, or lamp. A small portable resistance of novel construction is also shown, which, on being plugged to a lamp socket gives current suitable for galvanic and Faradic treatment. An induction coil giving a 12-inch spark was also on view. In connection with *x*-ray work a screen was shown surrounding the tube and protecting both operator and patient from the rays, whilst the area of the rays is limited to the diseased portion. Demonstrations with radium, with the *x*-rays, ultra-violet light, Finsen lamp, etc., were also given. The beds exhibited in the ward were three Lawson Tait beds fitted with the dominion mesh (Messrs. Gale and Co., of Birmingham, patent). One of the beds had a pulley, another was so constructed that by means of a handle fixed to the end of the bed, the patient can be raised into a sitting position. Round the third bed was a new hospital screen, the "Wreford," it is three-fold, with patent brass hinges, allowing the wings to be moved in any direction; by an ingenious arrangement of the frame and cover the screen can be drawn up to the middle of the bed ensuring privacy to the doctor and patient, the wings protecting the sides, and the middle the front of the bed. The advantage is that this can be done by means of one screen instead of two or three. Other appliances in the ward were an oxygen apparatus with oxygen warmer, a formalin disinfecting spray worked by gas from an oxygen cylinder, aseptic lockers, aseptic ward litter, electric bed warmer, batteries, a formalin fumigator, a powerful electric lamp for examination purposes, splints of all kinds, etc. The end room of the hospital was arranged as an ambulance section, containing ambulance requisites of all kinds. One portion of the hospital is fitted as a model *crèche* and has been furnished by C. Pratt and Sons, of Bradford.

